# The mediating role of education in ethnic disparities in late‐life cognition

**DOI:** 10.1002/alz70860_098182

**Published:** 2025-12-23

**Authors:** Judith J.M. Rijnhart, Ryan J Bailey, Jessica Agbodo, Vishakha Agrawal, Valerie M Rodriguez‐Olmo, Jason M Salemi

**Affiliations:** ^1^ University of South Florida, Tampa, FL, USA

## Abstract

**Background:**

Previous studies showed that, on average, Hispanic individuals have lower episodic memory scores compared to non‐Hispanic individuals. This ethnic disparity in episodic memory may be partially explained by differences in education across Hispanic and non‐Hispanic populations.

**Method:**

We used data on 19,623 community‐dwelling adults aged 51 and older who participated in the 2016 wave of the Health and Retirement study. Episodic memory was measured using immediate and delayed recall tests, with a higher score indicating better episodic memory (range 0‐20). We used the four‐way decomposition method to determine whether the ethnic disparity in episodic memory was driven by ethnic differences in the average number of education years, an interaction between ethnicity and education, or both. All effect estimates were controlled for confounding by gender, race, parental education, and birth year.

**Result:**

On average, Hispanic individuals had a 0.80 lower episodic memory score (95% confidence interval=‐1.01 to ‐0.60) compared to non‐Hispanic individuals. Effect estimates from the four‐way decomposition indicated that this ethnic disparity was partially driven by the lower average number of education years in the Hispanic subpopulation and the less beneficial impact of education on episodic memory in Hispanic individuals.

**Conclusion:**

To reduce ethnic disparities in episodic memory, ethnic differences in the average number of education years and the cognitive benefits of education need to be reduced. These findings highlight the importance of tailored interventions that target both access to education and the impact of education on episodic memory in Hispanic individuals in the United States.

